# COVID-19 Pandemic Responses among National Guard Service Members: Stressors, Coping Strategies, Sleep Difficultiesand Substance Use

**DOI:** 10.3390/ijerph20095731

**Published:** 2023-05-05

**Authors:** Holly B. Herberman Mash, Joshua C. Morganstein, Carol S. Fullerton, Robert J. Ursano

**Affiliations:** 1 Center for the Study of Traumatic Stress, Department of Psychiatry, Uniformed Services University of the Health Sciences, 4301 Jones Bridge Road, Bethesda, MD 20814, USA; joshua.morganstein@usuhs.edu (J.C.M.); carol.fullerton@usuhs.edu (C.S.F.); robert.ursano@usuhs.edu (R.J.U.); 2 Henry M. Jackson Foundation for the Advancement of Military Medicine, Inc., Bethesda, MD 20817, USA

**Keywords:** military, COVID-19, concerns, coping strategies, stress, substance use, sleep difficulties

## Abstract

The National Guard (NG) served as a critical component of the US COVID-19 response while concurrently managing personal COVID-19 responses. Understanding pandemic-related concerns, sleep difficulties, increased substance use, and stress management strategies can promote readiness for subsequent disasters. We surveyed 3221 NG service members (75% Army; 79% enlisted; 52% 30–49 years; 81% male) during COVID-19 (August-November 2020). Almost half were activated in response to COVID-19 (mean = 18.6 weeks) and completed the survey 2–3 months post-activation. Service members indicated great concern about family health (39%), the indefinite nature of the pandemic (35%), and their financial situation (23%). Over one-third reported changes in usual sleep amount, 33% described poor sleep quality, and 21% had trouble falling/staying asleep. Increased substance use was reported by 30%, including increased alcohol (13.5%), tobacco (9%), and caffeine/energy drinks (20.1%) consumption. Chi-square analyses and analyses of variance found those who activated reported more increased tobacco and caffeine/energy drink use versus non-activated, with no sleep difficulties nor alcohol use differences. Helpful stress management strategies included spending time outdoors (53%), exercising (48%), talking to family/friends (38%), and having a daily routine (38%). Specific health-, financial-, and job-related stressors were associated with COVID-19. Incorporating stress management in planning/preventive efforts promotes resilience during disasters.

## 1. Introduction

Since the beginning of 2020, the focus of the world has been on the extensive impact of the COVID-19 pandemic. This prolonged crisis has had a substantial adverse effect on the mental and behavioral health of individuals, and in particular, those who are responsible for responding to disasters. In the United States, National Guard (NG) service members are often deployed as first responders during national emergencies. The COVID-19 pandemic was no exception, and the NG has served as a critical component of the United States’ response. As with the rest of the Nation, NG service members have also been managing personal pandemic-related adversities and concerns. NG service members have experienced a unique combination of pandemic-related challenges, putting them at risk for adverse psychological and behavioral responses, such as sleep difficulties and increased substance use. To promote military readiness and provide support to NG service members, it is important to better understand their specific pandemic-related concerns, as well as strategies that they found helpful in managing disaster-related stress. In addition, identifying risk and protective factors associated with COVID-19 mental and behavioral health outcomes for both NG service members who were and were not activated in response to COVID-19 provides opportunities to identify targets for preventive interventions and may foster resilience.

Sleep difficulties are prevalent among some service members [1,2,3,4], with 16.4% of Army NG soldiers reporting moderate or severe levels of clinical insomnia [3]. Conditions specifically associated with deployment, including poor sleep environments and high operations tempo, may exacerbate sleep difficulties [2,5]. Sleep difficulties are also associated with psychological problems, impaired functioning and readiness, and mission-related errors [3,6,7]. Efforts to mitigate sleep difficulties and address high operational demands among military populations often result in increased caffeine/energy drink consumption [8], reported by one in six soldiers [9]. Similarly, problematic alcohol use has been identified in 16.5% of state NG soldiers [10], with a higher likelihood of alcohol problems identified among Army Reserve and National Guard (R/NG) soldiers as compared with those who are on active duty [11,12].

In the case of the COVID-19 pandemic, psychological and behavioral consequences have been substantial. Rates of US adults reporting increased mental health problems ranged from 40–56%, with 13% indicating that the pandemic was associated with initiating or increasing alcohol or substance use [13,14,15]. Further, several studies found increased sleep difficulties due to COVID-19 [16,17,18]. In a nationally representative US sample, 25% of individuals reported moderate to severe levels of insomnia during the pandemic [19]. First responders supporting COVID-19 efforts have been particularly vulnerable, with 7% of ICU staff working in nine hospitals in the United Kingdom reporting problem drinking during the summer of 2020 [20] and sleep problems identified among 31% of healthcare professionals [21]. Research has begun to examine responses to the COVID-19 pandemic among Veterans and military families. However, to our knowledge, studies have not yet been published that focus on military service members, and in particular the NG, the Armed Services component that provided the vast majority of military pandemic assistance. This underscores the importance of further assessment among NG service members who have and have not activated in response to COVID-19.

The current study examines the experiences of NG Unit (NGU) service members who provided support to a highly affected state, identified by the Centers for Disease Control and Prevention [22] as an early epicenter of the COVID-19 pandemic. We examined rates of sleep difficulties in the past month and increased substance consumption, specifically alcohol, tobacco, and caffeine/energy drink use. In addition, we report pandemic-related stressors that are unique to NGU service members and strategies that NG service members use to help manage pandemic-related stress. Understanding these outcomes may inform strategies to mitigate adverse psychological and behavioral responses, protect and prepare the force, and optimize readiness for additional waves of COVID-19 and similar future threats. 

## 2. Materials and Methods

### 2.1. Participants and Procedures

#### 2.1.1. Participants

A total of 3993 Army and Air Force state NGU service members participated in the study between August and December 2020, with the majority of survey participation (75%) completed by mid-October. Of these participants, 46% (*n* = 1363) activated prior to the data collection period (i.e., spring and summer 2020; mean activation length = 18.6 weeks). Activated NGU service members completed the survey approximately 2–3 months post-activation. Study respondents represented approximately 25% of the total NGU population (23% of the Army NG and 14% of the Air NG). 

Most participants were male (83.3%; *n* = 1220), white (67%; *n* = 776), and not married (60%; *n* = 768) (Table 1). The 30–39 year group was the modal age category (32%; *n* = 411). NGU participants were primarily Army NG (74.9%; *n* = 2414) and enlisted (79.2%; *n* = 2543), and similarly, those who activated were primarily Army NG (88.1%; *n* = 1297) and enlisted (80.2%; *n* = 1179).

#### 2.1.2. Procedures

Online survey invitations were distributed by email to all of the specific state NGU members. NGU service members were informed of the opportunity to participate in the study by their unit leaders via an email that provided a link to the survey. Survey instructions indicated that study participation was voluntary and anonymous. Service members completed the surveys on their personal computers or mobile phones and indicated agreement and consent to participate by completing the online survey. Participants were informed that the survey included items regarding their work and personal experiences during the COVID-19 pandemic. Personally identifiable information was not collected; all transmitted and stored data were non-identifiable. Survey completion took approximately 20 min. The study was conducted in accordance with the Declaration of Helsinki and approved on 26 June 2020 by the Institutional Review Board of the Uniformed Services University of the Health Sciences in Bethesda, Maryland (Protocol DBS.2020.125).

### 2.2. Measures 

#### 2.2.1. Health and Well-Being Concerns

Participants indicated the degree to which they experienced concern about 11 stressful experiences in the past four weeks, which included the following: (1) getting COVID-19; (2) spreading COVID-19 to others; (3) the indefinite nature of the pandemic; (4) being isolated from others; (5) your physical health; (6) your emotional health; (7) your spiritual health; (8) the health of your family; (9) your financial situation; (10) loss of your job; and (11) running out of food or supplies. These items were developed for the current study based on discussion with subject matter experts in disaster response and NG leadership. Response options included: (0) Not at all; (1) A little bit; (2) Moderate; (3) Quite a bit; and (4) Extreme. The degree to which participants found it difficult to balance their concerns for their family and their work/employment over the past four weeks was assessed, with response options ranging from (0) Not at all difficult to (4) Extremely difficult. We dichotomized scores for each concern item, using a cut-off of 3+ (Very or Extremely difficult) to indicate high levels of concern. A sum score of each of the dichotomized items was also created to identify the level of general concern for each NGU service member.

#### 2.2.2. Strategies for Managing Stress

Participants identified how helpful the following eight strategies were during the pandemic for managing stress: (1) talking to family or friends; (2) talking to mental health counselor; (3) community resources; (4) keeping to a daily routine; (5) exercise; (6) spending time outdoors; (7) religious/spiritual activities; and (8) activities online/computer/video games. Response options ranged from (0) Not at all to (4) Extremely. We dichotomized scores for each strategy item, using a cut-off of 3+ (Quite a Bit or Extremely helpful) to indicate high levels of helpfulness.

#### 2.2.3. Mental Health Outcomes

Sleep difficulties. Extent and quality of sleep difficulties over the past four weeks were assessed using three items. Change in sleep amount was assessed based on responses to the following item: “Compared to your usual amount of sleep, how much sleep have you had per night over the past four weeks?” Response options included: (1) Less than usual; (2) About the same; and (3) More than usual. Sleep quality was based on the item, “How would you rate your sleep quality overall in the past four weeks?” with response options ranging from (1) Very bad to (4) Very good. A third item measuring trouble falling asleep or staying asleep included response options ranging from (1) Much less trouble than usual to (5) Much more trouble than usual. 

#### 2.2.4. Increased Substance Use

Increased substance use, specifically alcohol, tobacco, and caffeine/energy drink use, for two weeks or more since the onset of the COVID-19 pandemic (approximately 8 months) was assessed using three separate items. Participants who responded that they either increased their use or had stopped but started using again were categorized with (1) increased use, and those who had decreased their use or use stayed the same were categorized with (0) no increased use. 

### 2.3. Statistical Analysis

Socio-demographics (age, gender, race, and marital status) and service-related characteristics (rank and military affiliation (Army, Air Force)), and COVID-19 activation were examined using descriptive statistics. Descriptive statistics also were used to identify rates of pandemic-related health and well-being concerns, strategies for managing stress, sleep difficulties in the past month, and increased alcohol, tobacco, and caffeine/energy drink use since the onset of COVID-19. Chi-square analyses and analyses of variance were conducted to examine the differences in concerns, strategies for managing stress, sleep difficulties, and increased substance use based on whether NGU participants were activated in response to COVID-19. Statistical analyses were conducted using SPSS software Version 28 [23]. 

## 3. Results

### 3.1. Health and Well-Being Concerns

Among all NGU service members in the past four weeks, they reported being most concerned about (1) the health of their family (with 39.2% reporting that they were quite a bit or extremely concerned); (2) the indefinite nature of the pandemic (35.0%); (3) their financial situation (23.2%); (4) their physical health (21.1%); and (5) spreading COVID-19 to others (20.2%) (See Table 1 for all categories). 

There was not a significant difference in the number of items about which NGU service members were highly concerned based on whether they activated (M(SD) = 2.25(2.92)) or did not activate (M(SD) = 2.36(2.90)). However, there were differences in rates of specific concerns based on activation status (Table 2). Chi-square analyses indicated that, as compared to NGU service members who activated, those who did *not* activate were more concerned about getting COVID-19 (19.0% of those who did not activate versus 15.5% of those who activated; χ^2^ = 6.28, *p* ≤ 0.01); the indefinite nature of the pandemic (36.7% versus 32.9%; χ^2^ = 4.60, *p* ≤ 0.05); being isolated from others (20.7% versus 15.0%, χ^2^ = 16.31, *p* ≤ 0.001); and the health of their family (41.5% versus 36.2%; χ^2^ = 8.61, *p* ≤ 0.01). Those who activated were more concerned about their financial situation than those who did not activate (25.5% of those who activated versus 21.3% of those who did not; χ^2^ = 7.40, *p* ≤ 0.01); and the loss of their job (15.6% versus 13.3%, χ^2^ = 2.98, *p* ≤ 0.05). 

As a result of the pandemic, 5.5% (*n* = 175) of all NGU service members reported permanently losing their jobs, and 8.8% (*n* = 284) temporarily losing their jobs or being furloughed. Over 14% (*n* = 400) indicated that it was very or extremely difficult to balance their concerns for their families and their work, with this balance reported as more difficult among NGU members who did *not* activate (16.3%) versus those who did (11.6%; χ^2^ = 12.61; *p* ≤ 0.001).

### 3.2. Strategies for Managing Stress

The top four strategies reported by all NGU service members as very or extremely helpful in managing stress during the COVID-19 pandemic were (1) spending time outdoors (52.9%); (2) exercising (47.8%); (3) talking to family or friends (38.3%); and (4) keeping to a daily routine (37.7%) (See Table 3 for all categories). There were differences in the helpfulness of strategies for managing stress among activation groups, with NGU service members who activated indicating that exercise (50.2% of those who activated versus 45.9% of those who did not; χ^2^ = 5.49, *p* ≤ 0.01); talking to family/friends (42.9% versus 34.6%; χ^2^ = 21.64, *p* ≤ 0.001); religious/spiritual activities (20.8% versus 16.8%; χ^2^ = 7.60, *p* ≤ 0.01); and community resources (10.4% versus 6.1%; χ^2^ = 18.42, *p* ≤ 0.001) were more helpful in managing stress than those who did not activate.

### 3.3. Mental and Behavioral Health

#### 3.3.1. Sleep Difficulties

Approximately 35% of all NGU service members (35.%; *n* = 989) indicated that they experienced a change in their usual amount of sleep in the past four weeks, with 30.3% (*n* = 846) reporting that they slept less than usual and 5.1% (*n* = 143) sleeping more than usual (Table 3). One-third (*n* = 931) reported that their sleep quality was fairly bad or very bad, and 21.3% (*n* = 597) indicated that they had more trouble falling asleep or staying asleep during this period. There were no significant differences in any of the sleep difficulty characteristics based on whether participants activated in response to COVID-19.

#### 3.3.2. Increased Substance Use

Among all participants, 30% (*n* = 839) reported an increase in at least one form of substance use for two weeks or more since the onset of the COVID-19 pandemic (Table 3). Specifically, 13.5% (*n* = 377) of all participants described an increase in alcohol use (and among those who reported drinking alcohol, 23.4% reported increased use), 9.0% (*n* = 251) described increased tobacco use (with 33.9% who smoke reporting increased use), and 20.1% (*n* = 562) reported increased caffeine/energy drink use (with 26.8% of those who use caffeine/energy drinks reporting increased use). Although there were no significant differences in increased alcohol use based on activation status, NGU service members who activated reported higher rates of increased tobacco use (10.9% of those who activated versus 7.4% who did not; χ^2^ = 10.27, *p* ≤ 0.001) and increased caffeine/energy drink use (22.7% of those who activated versus 17.9% who did not; χ^2^ = 9.72, *p* ≤ 0.001). When all substances were considered together, activated NGU service members reported a higher rate of increased use of any substance compared to those who had not activated (31.9% versus 28.4%, respectively; χ^2^ = 3.84, *p* ≤ 0.05).

## 4. Discussion

In addition to their roles as first responders during the pandemic, NG service members are vulnerable to the same life disruptions related to COVID-19 as civilians. As a result, NG service members who are, and even those who are not, activated in response to COVID-19 face increased COVID-19 exposure and other health risks while they concurrently manage concerns related to civilian employment, personal finances, and family safety. These concerns may strain familial/interpersonal relationships, adversely affect psychological, behavioral, and physical health, and influence force readiness and functioning. In this study, NGU service members were most concerned about the health of their families, the indefinite nature of the pandemic, their physical health, and the spreading of COVID-19 to others. This study also identified strategies that service members identified as helpful in managing stress, including spending time outdoors, exercising, talking to family or friends, and keeping to a daily routine. Programs that promote these behaviors can help reduce stress and adverse psychological and behavioral outcomes. 

The current study found that, in the past month, 30.3% of NGU service members reported sleeping less than usual, one-third reported poor sleep quality, and 21.3% indicated that they had more trouble falling or staying asleep. These rates were slightly elevated relative to those found in a nationally representative study of civilians during the pandemic [24], indicating that 19% of adults had trouble sleeping, with increased risk in females, consumption of high levels of alcohol use (i.e., >6 alcohol beverages per week), and reported stress [18,19,25,26]. The unique stressors of the NG population during the pandemic appear to have a particular impact on sleep for a notable proportion of service members. For NG and active duty Army soldiers, support resources such as leadership behaviors focused on promoting sleep (i.e., “sleep leadership” [7] can be effective management tools, increasing safety and reducing stress, mission-related errors, and burnout among unit members and other first responders [4,6].

In the current study, whether NGU service members activated in support of COVID-19 did not affect the likelihood of sleep difficulties. These findings correspond with previous research indicating that never-deployed US Army R/NG soldiers are at similar risk of negative mental health outcomes as those who had deployed [27,28]. However, those who have never deployed may face feelings of guilt and decreased connectedness, camaraderie, and value [29]. Crisis events, such as a pandemic or war, may also create a “stress on the force” that impacts all service members, regardless of their occupational responsibilities or exposures. Additional research that focuses on mental and behavioral health outcomes and specific types of sleep difficulties of NG service members during disasters, including those who are and are not activated, is warranted. 

Almost one-third (30%) of NGU service members reported an increase in at least one form of substance use for two weeks or more since the onset of the pandemic. Specifically, in the total sample, 13.5% increased alcohol use, 9% increased tobacco use, and 20.1% increased caffeine/energy drink use). Increased use appeared to be a greater issue among NGU service members during the pandemic as compared with civilians, among whom 8% reported increased alcohol or substance use during this period [24]. Given the noteworthy rate of problematic alcohol use generally found in a previous systematic review of R/NG service members (14.5%) [11], the increase in alcohol consumption associated with the pandemic among this population suggests that focus on problematic alcohol use merits attention. As with sleep difficulties, the lack of difference in increased alcohol use between NGU service members who did and did not activate may reflect the extent to which the pandemic touched everyone, regardless of activation status. Interestingly, however, those who had activated in response to COVID-19 reported higher rates of tobacco and caffeine/energy drink use as compared with those who did not activate. Increased tobacco use in those who smoke (found among 33.9% of smokers) may reflect an effort to manage stressors associated with activation [30,31]. Further, increased consumption of caffeine/energy drinks in those who were activated may be used in an effort for NG service members to remain alert for extended working hours and under periods of high stress. It also may be associated with NG service members’ attempts to contend with fatigue associated with disrupted sleep. However, substance use can exacerbate psychological and behavioral problems, including sleep difficulties [32,33,34]. These findings suggest that interventions that address substance use and encourage more adaptive health behaviors, such as sleep hygiene, among NG service members during the pandemic and other disasters may be important tools in maintaining behavioral and psychological health and promoting operational sustainment. 

Several limitations should be considered in the interpretation of the study findings. This study focuses on an important population of NG service members and can directly inform research on the NG and other first responders. However, participants consisted of NGU members in one state, and the generalization of study findings to other military and civilian populations may be limited and requires further study. In addition, the higher proportion of males in the study sample may limit generalizability to wider populations. It is also important for future research to consider the type of work tasks in which activated service members are engaged, particularly those associated with high-stress work conditions, to help better understand their activation experience and how it may influence post-activation responses. Further, recognizing the influence of NGU service members’ personal exposure to COVID-19, both their own and that of their families, provides additional insight into the impact of the activation experience on psychological and behavioral responses.

## 5. Conclusions

Screening for and being alert to service members who may be experiencing increased or problematic substance use and sleep difficulties can help identify those for whom to target preventive interventions. Additional research that focuses specifically on the influence of particular disaster-related concerns and strategies for managing stress in this population is important. The use of assessments and establishing training and interventions that directly address risk and protective factors may help prevent adverse psychological and behavioral responses, foster recovery, and promote force readiness.

## Figures and Tables

**Table 1:** Demographics and service-related characteristics and psychological and behavioral responses of National Guard Unit (NGU) service members.

	Activated NGU Service Members (*n* = 1363) ^a^		Not Activated NGU Service Members (*n* = 1735) ^a^		Total NGU Service Members (*n* = 3993)	
	*n*	%	*n*	%	*n*	%
**Demographics**						
**Gender**						
Men	1134	83.8	1374	79.7	2605	81.4
Women	219	16.2	349	20.3	596	18.6
**Race**						
White	768	67.0	1130	80.9	1906	74.6
Non-white	379	33.0	267	19.1	850	25.4
**Marital status**						
Not married	761	60.1	653	42.6	1421	50.5
Married	505	39.9	881	57.4	1392	49.5
**Age**						
<25 years	294	23.1	206	13.4	501	17.7
25–29 years	302	23.8	228	14.8	534	18.9
30–39 years	405	31.9	533	34.6	944	33.4
40+ years	269	21.2	573	37.2	845	29.9
**Service-Related Characteristics**						
**Military Affiliation**						
Army National Guard	1202	88.4	1105	63.7		
Air National Guard	157	11.6	630	36.3	807	25.1
**Rank**					2414	74.9
Enlisted	1086	80.0	1352	78.3	2543	79.2
Officer	272	20.0	374	21.7	668	20.8

^a^ *n*s in Activation group categories determined by participants who responded to the item (i.e., may not include the total sample of *n* = 3993).

**Table 2:** Health and well-being concerns and strategies for managing stress of National Guard Unit (NGU) service members.

	Activated NGU Service Members (*n* = 1363) ^a^		Not Activated NGU Service Members (*n* = 1735) ^a^		Total NGU Service Members (*n* = 3993)	
	*n*	%	*n*	%	*n*	%
**Health and Well-Being Concerns**						
**Getting COVID-19**						
Low	1121	84.5	1327	81.0	2448	82.5
High	206	15.5	312	19.0	518	17.5
**Spreading COVID-19 to Others**						
Low	1063	79.9	1308	79.8	2371	79.8
High	267	20.1	332	20.2	599	20.2
**Indefinite Nature of the Pandemic**						
Low	892	67.1	1038	63.3	1930	65.0
High	438	32.9	602	36.7	1040	35.0
**Being Isolated from Others**						
Low	1129	85.0	1300	79.3	2429	81.8
High	199	15.0	340	20.7	539	18.2
**Physical Health**						
Low	1046	78.8	1294	79.0	2340	78.9
High	281	21.2	345	21.0	626	21.1
**Emotional Health**						
Low	1081	81.3	1307	79.6	2388	80.4
High	248	18.7	334	20.4	582	19.6
**Spiritual Health**						
Low	1128	84.9	1408	85.9	2536	85.5
High	200	15.1	231	14.1	431	14.5
**Health of Family**						
Low	846	63.8	960	58.5	1806	60.8
High	481	36.2	682	41.5	1163	39.2
**Financial Situation**						
Low	988	74.5	1289	78.7	2277	76.8
High	339	25.5	349	21.3	688	23.2
**Loss of Job**						
Low	1116	84.4	1422	86.7	2538	85.7
High	206	15.6	219	13.3	425	14.3
**Running Out of Food or Supplies**						
Low	1200	90.4	1489	90.7	2689	90.6
High	127	9.6	152	9.3	279	9.4
**Helpfulness of Stress Management Strategies**						
**Talking to Family/Friends**						
No	758	57.1	1073	65.4	1831	61.7
Yes	570	42.9	567	34.6	1137	38.3
**Talking to Mental Health Counselor**						
No	1228	92.7	1533	93.6	2761	93.2
Yes	97	7.3	104	6.4	201	6.8
**Community Resources**						
No	1186	89.6	1536	93.9	2722	92.0
Yes	137	10.4	99	6.1	236	8.0
**Keeping to a Daily Routine**						
No	811	61.1	1033	63.2	1844	62.3
Yes	517	38.9	601	36.8	1118	37.7
**Exercise**						
No	660	49.8	884	54.1	1544	52.2
Yes	666	50.2	750	45.9	1416	47.8
**Spending Time Outdoors**						
No	629	47.4	769	46.8	1398	47.1
Yes	699	52.6	873	53.2	1572	52.9
**Religious/Spiritual Activities**						
No	1050	79.2	1362	83.2	2412	81.4
Yes	276	20.8	276	16.8	552	18.6
**Activities Online/Computer/Video Games**						
No	910	68.9	1230	75.3	2140	72.4
Yes	411	31.1	403	24.7	814	27.6

^a^ *n*s in Activation group categories determined by participants who responded to the item (i.e., may not include the total sample of *n* = 3993).

**Table 3:** Mental and behavioral health of National Guard Unit (NGU) service members.

	Activated NGU Service Members (*n* = 1363) ^a^		Not Activated NGU Service Members (*n*= 1735) ^a^		Total NGU Service Members (*n* = 3993)	
	*n*	%	*n*	%	*n*	%
**Sleep Difficulties (Past 4 weeks)**						
**Sleep Amount**						
Less than usual	382	30.0	464	30.4	846	30.3
About the same	819	64.4	988	64.8	1807	64.6
More than usual	71	5.6	72	4.7	143	5.1
**Sleep Quality**						
Fairly good to very good	864	67.9	1003	65.7	1867	66.7
Fairly bad to very bad	408	32.1	523	34.3	931	33.3
**Trouble Falling or Staying Asleep**						
Low	1001	78.9	1196	78.4	2197	78.6
High	267	21.1	330	21.6	597	21.4
**Increased Substance Use (2+ weeks since COVID-19 onset)**						
**Any Increased Substance Use**						
No	864	68.1	1094	71.6	1958	70.0
Yes	404	31.9	435	28.4	839	30.0
**Increased Alcohol Use**						
No	1109	87.2	1316	86.0	2425	86.5
Yes	163	12.8	214	14.0	377	13.5
**Increased Tobacco Use**						
No	1131	89.1	1415	92.6	2546	91.0
Yes	138	10.9	113	7.4	251	9.0
**Increased Caffeine/Energy Drink Use**						
No	983	77.3	1255	82.1	2238	79.9
Yes	288	22.7	274	17.9	562	20.1

^a^ *n*s in Activation group categories determined by participants who responded to the item (i.e., may not include the total sample of *n* = 3993).

## Data Availability

The data presented in this study are available on request from the corresponding author. The data are not publicly available due to privacy restrictions.

## References

[1] Adler A.B., Bliese P.D., McGurk D., Hoge C.W., Castro C.A. (2009). Battlemind debriefing and battlemind training as early interventions with soldiers returning from Iraq: Randomization by platoon. J. Consult. Clin. Psychol..

[2] Gunia B.C., Sipos M.L., LoPresti M., Adler A.B. (2015). Sleep leadership in high-risk occupations: An investigation of soldiers on peacekeeping and combat missions. Mil. Psychol..

[3] Hansen L.P., Kinskey C., Koffel E., Polusny M., Ferguson J., Schmer-Galunder S., Erbes C.R. (2018). Sleep patterns and problems among Army National Guard soldiers. Mil. Med..

[4] Sianoja M., Crain T.L., Hammer L.B., Bodner T., Brockwood K.J., LoPresti M., Shea S.A. (2020). The relationship between leadership support and employee sleep. J. Occup. Health Psychol..

[5] Peterson A.L., Goodie J.L., Satterfield W.A., Brim W.L. (2008). Sleep disturbance during military deployment. Mil. Med..

[6] Adler A.B., Bliese P.D., LoPresti M.L., McDonald J.L., Merrill J.C. (2021). Sleep leadership in the Army: A group randomized trial. Sleep Health.

[7] LoPresti M.L., Anderson J.A., Saboe K.N., McGurk D., Balkin T.J., Sipos M.L. (2016). The impact of insufficient sleep on combat mission performance. Mil. Behav. Health.

[8] McLellan T.M., Riviere L.A., Williams K.W., McGurk D., Lieberman H.R. (2019). Caffeine and energy drink use by combat arms soldiers in Afghanistan as a countermeasure for sleep loss and high operational demands. Nutr. Neurosci..

[9] Toblin R.L., Adrian A.L., Hoge C.W., Adler A.B. (2018). Energy drink use in U.S. service members after deployment: Associations with mental health problems, aggression, and fatigue. Mil. Med..

[10] Coughlin L.N., Walton M.A., McCormick R., Blow F.C. (2019). Prevalence and severity of alcohol and cannabis use across the urban-rural continuum in the Michigan National Guard. J. Rural Health.

[11] Cohen G.H., Fink D.S., Sampson L., Galea S. (2015). Mental health among reserve component military service members and veterans. Epidemiol. Rev..

[12] Hoopsick R.A., Homish D.L., Vest B.M., Bartone P.T., Homish G.G. (2021). Resilience to hazardous drinking among never-deployed male United States Army Reserve and National Guard soldiers. Alcohol. Clin. Exp. Res..

[13] Han R.H., Schmidt M.N., Waits W.M., Bell A.K.C., Miller T.L. (2020). Planning for mental health needs during COVID-19. Curr. Psychiatry Rep..

[14] Kirzinger A., Hamel L., Muñana C., Kearney A., Brodie M. KFF Health Tracking Poll—Late April 2020: Coronavirus, Social Distancing, and Contact Tracing. https://www.kff.org/coronavirus-covid-19/issue-brief/kff-health-tracking-poll-late-april-2020/.

[15] Czeisler M.É., Lane R.I., Petrosky E., Wiley J.F., Christensen A., Njai R., Weaver M.D., Robbins R., Facer-Childs E.R., Barger L.K. (2020). Mental health, substance use, and suicidal ideation during the COVID-19 pandemic—United States, June 24–30. MMWR.

[16] McCarthy E., DeViva J.C., Na P.J., Pietrzak R.H. (2021). New-onset and exacerbated insomnia symptoms during the COVID-19 pandemic in U.S. military veterans: A nationally representative, prospective cohort study. J. Sleep Res..

[17] Wu T., Jia X., Shi H., Niu J., Yin X., Xie J., Wang X. (2021). Prevalence of mental health problems during the COVID-19 pandemic: A systematic review and meta-analysis. J. Affect. Disord..

[18] Yuksel D., McKee G.B., Perrin P.B., Alzueta E., Caffarra S., Ramos-Usuga D., Arango-Lasprilla J.C., Baker F.C. (2021). Sleeping when the world locks down: Correlates of sleep health during the COVID-19 pandemic across 59 countries. Sleep Health.

[19] Killgore W.D.S., Cloonan S.A., Taylor E.C., Fernandez F., Grandner M.A., Dailey N.S. (2020). Suicidal ideation during the COVID-19 pandemic: The role of insomnia. Psychiatry Res..

[20] Greenberg N., Weston D., Hall C., Caulfield T., Williamson V., Fong K. (2021). Mental health of staff working in intensive care during COVID-19. Occup. Med..

[21] Alimoradi Z., Brostrom A., Tsang H.W.H., Griffiths M.D., Haghayegh S., Ohayon M.M., Lin C.-Y., Pakpour A.H. (2021). Sleep problems during COVID-19 pandemic and its association to psychological distress: A systematic review and meta-analysis. eClin. Med. Lancet.

[22] Centers for Disease Control and Prevention (2020). COVID-19 Outbreak—New York City, February 29–June 1. MMWR.

[23] IBM Corp (2021). IBM SPSS Statistics for Windows, Version 28.

[24] American Psychiatric Association New Poll: COVID-19 Impacting Mental Well-Being: Americans Feeling Anxious, Especially for Loved Ones; Older Adults Are Less Anxious. https://www.psychiatry.org/newsroom/news-releases/new-poll-covid-19-impacting-mental-well-being-americans-feeling-anxious-especially-for-loved-ones-older-adults-are-less-anxious.

[25] Mandelkorn U., Genzer S., Choshen-Hillel S., Reiter J., Meira E., Cruz M., Hochner H., Kheirandish-Gozal L., Gozal D., Gileles-Hillel A. (2021). Escalation of sleep disturbances amid the COVID-19 pandemic: A cross-sectional international study. J. Clin. Sleep Med..

[26] Meaklim H., Junge M.F., Varma P., Finck W.A., Jackson M.L. (2021). Pre-existing and post-pandemic insomnia symptoms are associated with high levels of stress, anxiety and depression globally during the COVID-19 pandemic. J. Clin. Sleep Med..

[27] Hoopsick R.A., Homish D.L., Collins R.L., Nochajski T.H., Read J.P., Bartone P.T., Homish G.G. (2021). Resilience to mental health problems and the role of deployment status among U.S. Army Reserve and National Guard soldiers. Soc. Psychiatry Psychiatr. Epidemiol..

[28] Hoopsick R.A., Homish D.L., Collins R.L., Nochajski T.H., Read J.P., Homish G.G. (2020). Is deployment status the critical determinant of psychosocial problems among Reserve/Guard soldiers?. Psychol. Serv..

[29] Hoopsick R.A., Homish D.L., Bartone P.T., Homish G.G. (2018). Developing a measure to assess emotions associated with never being deployed. Mil. Med..

[30] Brown J.M., Goodell E.M.A., Williams J., Bray R.M. (2018). Socioecological risk and protective factors for smoking among active duty US military personnel. Mil. Med..

[31] Hermes E.D.A., Wells T.S., Smith B., Boyko E.J., Gackstetter G.G., Miller S.C., Smith T.C. (2011). Smokeless tobacco use related to military deployment, cigarettes, and mental health symptoms in a large, prospective cohort study among US service members. Addiction.

[32] Debell F., Fear N.T., Head M., Batt-Rawden S., Greenberg N., Wessely S., Goodwin L. (2014). A systematic review of the comorbidity between PTSD and alcohol misuse. Soc. Psychiatry Psychiatr. Epidemiol..

[33] McLean C.P., Zandberg L., Roache J.D., Fitzgerald H., Pruiksma K.E., Taylor D.J., Dondanville K.A., Litz B.T., Mintz J., Young-McCaughan S. (2019). Caffeine use in military personnel with PTSD: Prevalence and impact on sleep. Behav. Sleep Med..

[34] Stein M.D., Friedman P.D. (2005). Disturbed sleep and its relationship to alcohol use. Subst. Abus..

